# Impact of Freeze–Thaw Cycles on Die-Off of *E. coli* and Intestinal Enterococci in Deer and Dairy Faeces: Implications for Landscape Contamination of Watercourses

**DOI:** 10.3390/ijerph17196999

**Published:** 2020-09-24

**Authors:** Emmanuel O. Afolabi, Richard S. Quilliam, David M. Oliver

**Affiliations:** Biological & Environmental Sciences, Faculty of Natural Sciences, University of Stirling, Stirling FK9 4LA, UK; richard.quilliam@stir.ac.uk (R.S.Q.); david.oliver@stir.ac.uk (D.M.O.)

**Keywords:** faecal pollution, indicator organisms, microbial contamination, land management, water quality, wildlife faeces

## Abstract

Characterising faecal indicator organism (FIO) survival in the environment is important for informing land management and minimising public health risk to downstream water users. However, key gaps in knowledge include understanding how wildlife contribute to catchment-wide FIO sources and how FIO survival is affected by low environmental temperatures. The aim of this study was to quantify *E. coli* and intestinal enterococci die-off in dairy cow versus red deer faecal sources exposed to repeated freeze–thaw cycles under controlled laboratory conditions. Survival of FIOs in water exposed to freeze–thaw was also investigated to help interpret survival responses. Both *E. coli* and intestinal enterococci were capable of surviving sub-freezing conditions with the faeces from both animals able to sustain relatively high FIO concentrations, as indicated by modelling, and observations revealing persistence in excess of 11 days and in some cases confirmed beyond 22 days. Die-off responses of deer-derived FIOs in both faeces and water exposed to low temperatures provide much needed information to enable better accounting of the varied catchment sources of faecal pollution and results from this study help constrain the parameterisation of die-off coefficients to better inform more integrated modelling and decision-making for microbial water quality management.

## 1. Introduction

Agricultural landscapes can harbour a large burden of faecal indicator organisms (FIOs), such as *Escherichia coli* and intestinal enterococci. Major contributors to this burden include grazing livestock and land applications of both solid and liquid manures [1,2]. Microbial water quality is inferred via FIO concentration but the detection of FIOs in water samples does not necessarily imply that pathogens are present; rather, increased concentrations of FIOs signal a higher level of faecal pollution [3]. Knowledge of how FIOs survive in the environment is therefore important for informing land management and understanding wider aspects of public health risk to downstream water users, e.g., those exposed to contaminated recreational water [4]. However, there is now a growing recognition that wildlife, e.g., deer and geese, can further contribute to the FIO burden in rural and agricultural landscapes [5]; the importance of this contribution to downstream impacts on microbial water quality is relatively unknown [6].

FIO survival outside of the host gut is strongly influenced by temperature [7]. Previous research has focused on FIO persistence under constant temperature conditions and how diurnal temperature fluctuations can impact on FIO survival [8,9], with particular attention given to the likely effects of climate change and warming temperature cycles on the persistence profiles of faecal bacteria (e.g., [10,11]). There are, however, relatively few studies of FIO survival at low environmental temperatures, including subfreezing conditions, or through freeze–thaw (F–T) cycles, and those that do exist have focused on FIOs in soil and water matrices. Findings from those studies have identified reduced *E. coli* survival times in river water undergoing repeated F–T stress, with a more pronounced reduction in cell numbers during the first F–T cycle [12]. Similarly, there are reports of repeated F–T cycles in soil accelerating die-off rates of enteric bacteria relative to constant cold temperature conditions [13,14] and total coliforms have been found to persist in excess of six months in subfreezing soil temperatures [15]. Bacterial cells that enter the soil pore architecture after mobilisation from faeces are likely to be more susceptible to freezing conditions than those that remain in the protective insulation and nutrient rich matrix of a faecal deposit, but data to confirm or refute this are lacking.

Overprediction of modelled versus observed *E. coli* burden at the landscape scale during winter has been hypothesised to be a consequence of non-conducive conditions of sub-freezing temperatures for *E. coli* survival [16]. Indeed, many catchment scale models of FIO fate and transfer are highly parameterised to account for typical seasonal temperature effects on cell persistence (e.g., [17]) and yet the impact of sub-zero temperatures and F–T on FIO concentrations in faeces remains largely unquantified and missing from such models [18,19]. This is in parallel to the lack of inclusion of relevant die-off coefficients for FIOs derived from wildlife faeces in general. Understanding whether FIOs are insulated from F–T processes by a protective faecal matrix and quantifying the impacts, if any, on FIO die-off rates for different F–T temperature regimes is therefore important to more fully account for the temporal dynamics of FIO burden in the landscape. In addition to the effects of varying F–T temperatures on FIO survival, there is also likely to be differential protection of the FIO population attributed to the characteristics of the faecal source, e.g., faecal pats versus faecal pellets.

Opportunities for livestock and wildlife faecal deposits to undergo F–T stress are not uncommon. While cattle may be offered some protection from cold weather in the form of housing, European temperate grassland management sometimes favours early turnout of cows to pasture in spring when overnight temperatures can promote F–T, and in other areas of the world, e.g., New Zealand, the use of cattle housing, and in turn protection from F–T, is much less common than in the UK or the USA [20]. Extensive sheep grazing in remote uplands is also typical in many regions of the world where both livestock and wildlife faeces will be frequently exposed to regular F–T processes during colder seasons of the year and indeed other seasons depending on altitude.

The overarching aim of this study, therefore, was to characterise *E. coli* and intestinal enterococci die-off in dairy cow versus red deer faecal sources exposed to F–T cycles representative of environmental conditions during the colder seasons of temperate regions. The specific objectives of the experiment were to: (i) quantify differences in die-off of FIOs exposed to varying degrees of F–T cycling relative to faeces held at constant low temperatures; (ii) evaluate whether the nature of the faecal source influenced the rate of die-off observed during F–T cycles relative to freely suspended cells in water; and (iii) provide parameter values to represent new understanding of the importance of F–T processes influencing the environmental persistence of FIOs to better inform more integrated modelling and decision-making for microbial water quality management.

## 2. Materials and Methods

### 2.1. Provenance of Faeces Used in All Experiments

Fresh dairy faeces were collected from the livestock housing of a conventional dairy farm in Stirlingshire, Scotland. Cows were permanently housed and a mechanical barn floor scraper was in operation meaning that any faeces collected was guaranteed to have been deposited within the previous 30 min. Fresh faeces of red deer were collected from the Scottish Deer Centre, Fife, Scotland. Fields containing deer were harrowed prior to faecal collection, which ensured that all faeces collected were fresh (<12 h old). After collection, all faeces were transferred immediately (<1 h) to the laboratory for use in the experiment and thus no interim storage was required.

### 2.2. Experiment Design

#### 2.2.1. Faecal Mesocosms

A laboratory-controlled experiment was used to mimic the effect of F–T temperature cycles on the survival of FIOs indigenous in red deer and dairy cow faeces. All experiments were carried out in temperature-programmable incubators (Sanyo Incubator MIR−153, Japan). Two temperature treatments cycling over 24 h periods were used: (i) 4 °C, 0 °C, −4 °C (herein −4 °C F–T); and (ii) 0 °C, −4 °C, −8 °C (herein −8 °C F–T). Both treatments spanned an 8 °C temperature range to focus investigation on different temperature extremes rather than rates of temperature change. Faeces were held for 8 h at each of the three temperatures during the 24 h cycle to ensure a rapid thaw and a gradual freeze. Two constant temperature control treatments were used (4 °C for comparison with the −4 °C F–T treatment and 0 °C for comparison with the −8 °C treatment). Both treatments ran for a minimum of 11 days and maximum of 22 days. Each F–T and control temperature treatment consisted of five and three replicate faecal pat/pellet deposits per sampling day, respectively. The use of full-size faecal deposits was impractical for a replicated laboratory experiment; therefore, faecal samples were bulked and homogenised in a sterile plastic container and then distributed into shallow circular 70 mm diameter foil trays as either 100 g dairy faecal deposits or 90 g piles of deer faecal pellets. Replicates were randomly divided into each treatment and each replicate was destructively sampled on days 0, 1, 2, 4, 7, 11 and (where relevant) day 22. All treatments included two additional faecal samples that were used to measure the internal temperature of faeces over the course of the experiment, i.e., a DS1921G Thermochron i-button temperature logger (iButtonLink; Whitewater, WI, USA) was placed within the core of the faecal matrix. The same loggers were used as a quality control indicator of the incubator air temperature; in some treatments a drift in the F–T temperature regime was observed after ~ 14 days and in those cases, any data derived after sampling day 11 were not used. Every two days all faecal deposits were misted with sterile distilled water at a rate of 1 mL/100 cm^2^ to avoid complete dehydration of the faeces under incubator conditions. Prior to sampling, each replicate faecal deposit was weighed to determine fresh weight change over time. Next, approximately 30 g of faeces (6 × 5 g subsamples) was randomly sampled from each replicate tray using a sterile spatula and transferred to a sterile 50 mL collection tube. Faecal samples were extracted from the core of the deposits to avoid sampling surface crust. Microbial analysis to determine concentrations of colony forming units (CFU) was initiated immediately after obtaining the samples.

#### 2.2.2. Water Mesocosms

In order to investigate whether a faecal matrix offered protection from F–T impacts, waterborne FIOs were subjected to the same F–T conditions. Plastic tubes contained 40 mL of sterile distilled water and were inoculated with 1 mL of a mixed inoculant of *E. coli* and intestinal enterococci with an initial concentration of ~8.0 and 7.2 log_10_ CFU/mL, respectively. The inoculant was prepared from *E. coli* and intestinal enterococci strains isolated from either the dairy or deer faeces from the same herds. Cells from an overnight culture of LB broth (Fisher Bioreagents, UK) were harvested following centrifugation at 3600 rpm for 3 min. The pelleted cells were resuspended in 10 mL phosphate buffered saline (PBS, Fisher Bioreagents, NJ, USA) and washed three times through resuspension and centrifugation before final suspension in 40 mL PBS. Three replicates per sampling day were randomly divided into each F–T treatment and replicates was destructively sampled on the same days as the respective faecal treatments.

### 2.3. FIO Enumeration

At each time point, approximately one gram of faeces was transferred to 9 mL of sterile PBS in a 15 mL centrifuge tube and homogenised using an orbital shaker (160 rpm for 60 min at ambient temperature). Each tube was vortex mixed for 30 s prior to subsequent 1:10 serial dilution in PBS. For water samples, 1 mL of water from each replicate mesocosm was transferred to 9 mL of sterile PBS and vortex mixed for 30 s prior to subsequent 1:10 serial dilution in PBS.

Briefly, 1 mL of each serially diluted sample was pipetted onto a sterile 0.45 µm cellulose acetate membrane and washed through a vacuum-filtration unit (Sartorius Stedim Biotech., Goettingen, Germany) with ~20 mL of sterile PBS. To determine presumptive *E. coli,* membranes were aseptically transferred to a Petri dish containing Membrane Lactose Glucuronide Agar (MLGA) (CM1031, Oxoid, Basingstoke, UK) and incubated inverted at 37 °C (±0.2 °C) for 18–24 h. To quantify intestinal enterococci, membranes were aseptically transferred to Slanetz and Bartley medium (CM0377, Oxoid) and incubated inverted at 44 °C (±0.2 °C) for 48 h. A spread plate method was also used where necessary to complement filtration techniques, e.g., at low serial dilutions to avoid interference of faecal particles with CFU growth on membrane filters. Intestinal enterococci isolates were aseptically transferred to Kanamycin Sulphate Supplement agar and incubated for 6 h at 37 °C to confirm that they were of faecal rather than environmental origin: all isolates were confirmed as faecal. Method blanks (i.e., sterile PBS) were used to confirm aseptic technique and the flame sterilisation procedure between samples. The limit of detection was 50 CFU per g fresh weight faeces. All sample analysis was performed in duplicate. The remaining faecal sample (~29 g) was used to determine the gravimetric water content by drying at 105 °C for 48 h (until constant mass) and weighing the residual to allow all FIO concentrations to be expressed as CFU g^−1^ dry weight of faeces.

### 2.4. Statistical Analysis

#### 2.4.1. Faecal Samples

Plate counts of *E. coli* and intestinal enterococci were normalised by transforming to log_10_ CFU g^−1^ dry weight faeces. For both FIOs, non-linear regression analysis was used to establish the relationship that best described the pattern of decline in each faecal source (dairy, deer) and temperature (−8 °C F–T, −4 °C F–T, 0 °C, 4 °C) treatment. An exponential model was fitted to each resulting time-series of FIO die-off associated with the five replicates of deer and dairy faecal samples exposed to two contrasting F–T temperature regimes. The exponential model fitted to the log_10_ transformed FIO data is described by Equation (1):

Log_10_(C) = A + Be^-λt^ (1)

where C is the cell concentration (CFU g^−1^), λ is the exponential rate of decline (d^−1^), governing the decay of the die-off rate constant over time, B is the difference in cell numbers between experiment start and finish (log_10_ CFU g^−1^), A is the final level of bacterial population stability (log_10_ CFU g^−1^) and t is time (d). The % decrease in FIO concentration per unit time is not constant and instead decays with time.

A three-way analysis of variance (ANOVA) and a Tukey multiple comparison test were used to test for differences in λ, A and B associated with the fitted models as a function of FIO type, faecal source and F–T temperature cycle and to test for any interactions between these factors (Minitab 18.0 software, Minitab Inc.; State College, PA, USA). The same exponential model was fitted to the control treatments and one-way ANOVA and Mann–Whitney U tests were used to test for differences in λ, A and B associated with the models of die-off for FIOs in the control versus F–T treatments. Differences at the *p* < 0.05 level (95% confidence interval) were considered statistically significant.

#### 2.4.2. Water Samples

Plate counts of *E. coli* and intestinal enterococci were normalised by transforming to log_10_ CFU mL^−1^. No single model was suitable for all die-off data and so linear and non-linear regression were used as appropriate to model FIO decline for each faecal source and temperature treatment. The log-linear model fitted to the log_10_ transformed FIO data is described by Equation (2):

Log_10_(C) = Log_10_(C_0_) − *k*t (2)

where C_0_ is the cell concentration at t = 0 and *k* is a die-off rate constant (d^−1^). This model describes die-off based on first-order kinetics whereby the % decrease in FIO concentration per unit time is constant. *D*-values, which represent decimal reduction times, were calculated based on the average rate of decline for those populations following a log-linear die-off profile. For those water treatments where a non-linear model was fitted to the data, a one-way ANOVA was used to test for differences in their die-off characteristics relative to the faecal treatments exposed to the same F–T temperature cycle.

#### 2.4.3. Interpreting Parameter Values of Log—Linear and Exponential Models

The % decrease in FIO population per unit time associated with the exponential model fitted to the log_10_ transformed FIO data jointly reflects λ and the difference in cell numbers over the experiment (parameter B). In this model λ is the decay rate constant of the die-off rate constant, describing how quickly the die-off rate constant decreases in time. Thus, higher λ equates to a more rapid decay of the rate constant; i.e., the % decrease (in FIO concentration) per unit time is initially high but rapidly declines. In contrast, *k* represents a die-off rate constant in the log-linear model and it alone directly sets the % decrease in concentration per unit time; i.e., higher *k* equates to a higher % decrease in population per unit time.

## 3. Results

### 3.1. FIO Die-Off in Faeces

**Figure 1:**
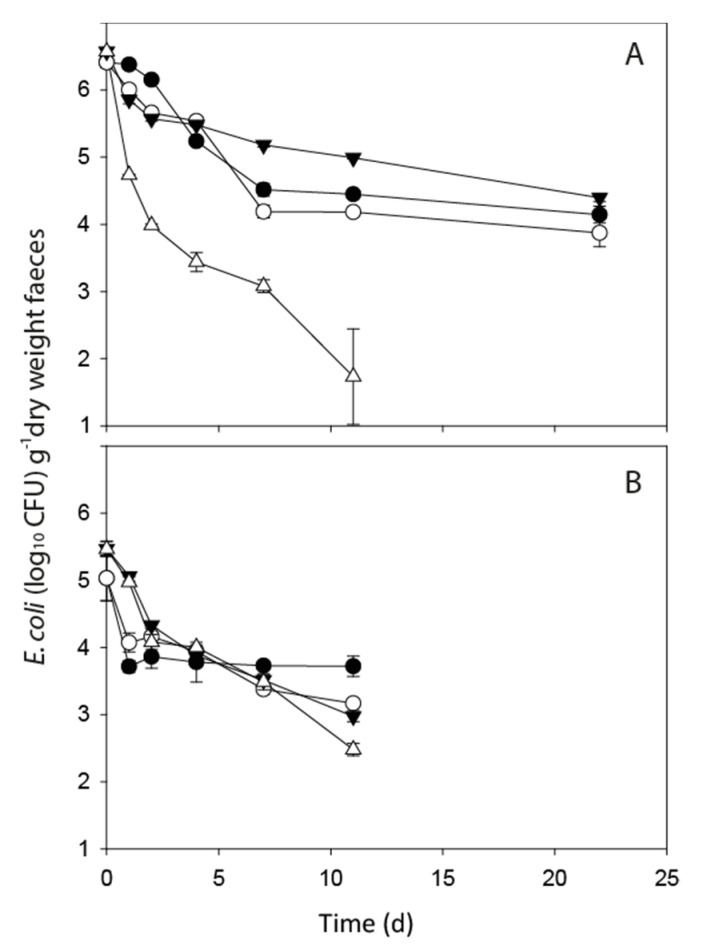
*E. coli* die-off profiles in dairy faeces (**A**) and deer faeces (**B**) held at: constant 4 ^°^C (solid black circle); −4 °C freeze-thaw (open circles); constant 0 °C (solid triangle); −8 °C freeze-thaw (open triangles). Data points are the mean of five replicates ± standard error (freeze–thaw) and three replicates ± standard error (constant).

**Figure 2:**
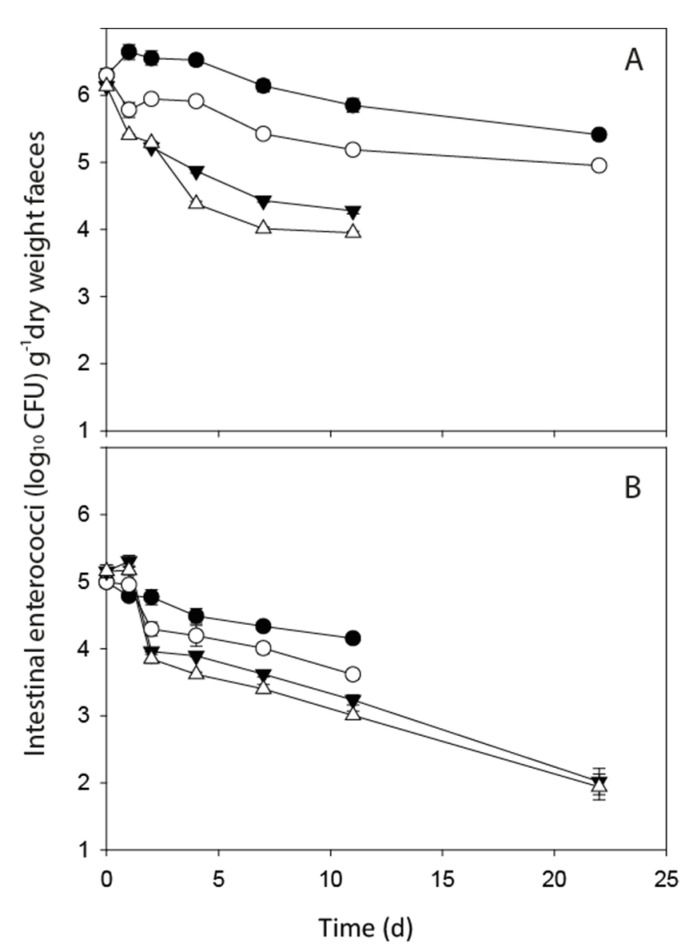
Intestinal enterococci die-off profiles in dairy faeces (**A**) and deer faeces (**B**) held at: constant 4 °C (solid black circle); −4 °C freeze–thaw (open circles); constant 0 °C (solid triangle); −8 °C freeze-thaw (open triangles). Data points are the mean of five replicates ± standard error (freeze–thaw) and three replicates ± standard error (constant).

No *E. coli* populations showed any growth immediately post-defecation (Figure 1). However, there was evidence of short-term cell growth in two of the intestinal enterococci treatments. In deer faeces held at 0 °C, there was a small increase of 0.15 log_10_ CFU g^−1^ dry weight faeces in the initial 24 h but this was followed by a drop of 1.34 log_10_ CFU g^−1^ dry weight faeces. In dairy faeces held at a constant 4 °C, an increase of 0.35 log_10_ CFU g^−1^ dry weight faeces was recorded and cell numbers were sustained longer than in the deer faeces (Figure 2A,B).

Mean FIO concentrations in fresh faeces from the dairy cow and red deer sources are shown in Table 1. The persistence profiles of *E. coli* and intestinal enterococci in deer and dairy faeces under different temperature regimes were recorded over a minimum of 11 days and over the timeframe of sampling all FIO concentrations decreased, with changes most pronounced during the initial 24 h (Figure 1 and Figure 2). Parameter results from the fitting of the non-linear model to all faecal treatments are shown in Table 2 (*E. coli*) and Table 3 (intestinal enterococci). Overall, no significant difference was recorded between the values of λ, the exponential rate constant, for *E. coli* or intestinal enterococci in dairy and deer faeces held at the two different F–T cycles. However, there was a significant interactive effect of faecal source and temperature on FIO exponential rate constants (*p* < 0.01). While there was a clear visual difference in the pattern of *E. coli* decline for the dairy faeces exposed to the −8 °C F–T, the high variability in *E. coli* numbers at day 11 resulted in no statistically significant difference in λ values across treatments (Figure 1A). On day 11, two of the five dairy replicates exposed to the −8 °C F–T treatment dropped below detection limits and there is clear variability in *E. coli* concentrations in the replicates as time increases. There was no significant difference between λ values determined for both FIOs exposed to −4 °C and −8 °C F–T cycles in both faecal types relative to the equivalent FIOs held at constant temperatures of 4 °C and 0 °C, respectively.

Three-way ANOVA did, however, identify significant differences in the non-linear model parameters A and B between treatments. For parameter A (the final concentration of population stability) significant differences were identified between faecal source (*p* < 0.01) and F–T temperature cycle (*p* < 0.001). Dairy faeces supported higher modelled final FIO concentrations than deer faeces and lower modelled levels of population stability were recorded for cells exposed to the lower temperature (−8 °C) F–T cycle. There were no significant interactions between factors. Parameter B represents the magnitude of population decline, removing the potential influence of differences in the initial concentration. For parameter B, a significant difference was identified between FIO type (*p* < 0.001) and F–T temperature cycle (*p* < 0.001), but not between faecal source (*p* > 0.05). Cells exposed to the −8 °C F–T cycle recorded the higher magnitude decline in cell numbers and *E. coli* recorded a higher decline relative to intestinal enterococci, consistent with findings for parameter A. In addition, a significant interaction occurred between FIO type and faecal source (*p* < 0.001) and FIO type and F–T temperature cycle (*p* < 0.001), but not between faecal source and F–T temperature cycle (*p* > 0.05).

In dairy faeces, no significant difference was determined for A and B values of survival curves for *E. coli* monitored at 4 °C versus a −4 °C F–T cycle. However, there was a significant difference for both parameters when comparing the 0 °C and −8 °C F–T cycle, with the final concentration of modelled *E. coli* population stability significantly lower for the F–T treatment relative to the constant 0 °C treatment, and so a larger population decline was also recorded for the F–T treatment (*p* < 0.05). The final concentration of population stability was significantly lower for intestinal enterococci exposed to the −8 °C F–T treatment relative to the 0 °C treatment. Differences in parameters A and B were not evident in the modelled survival curves for *E. coli* in deer faeces.

### 3.2. FIO Die-Off in Water

**Figure 3:**
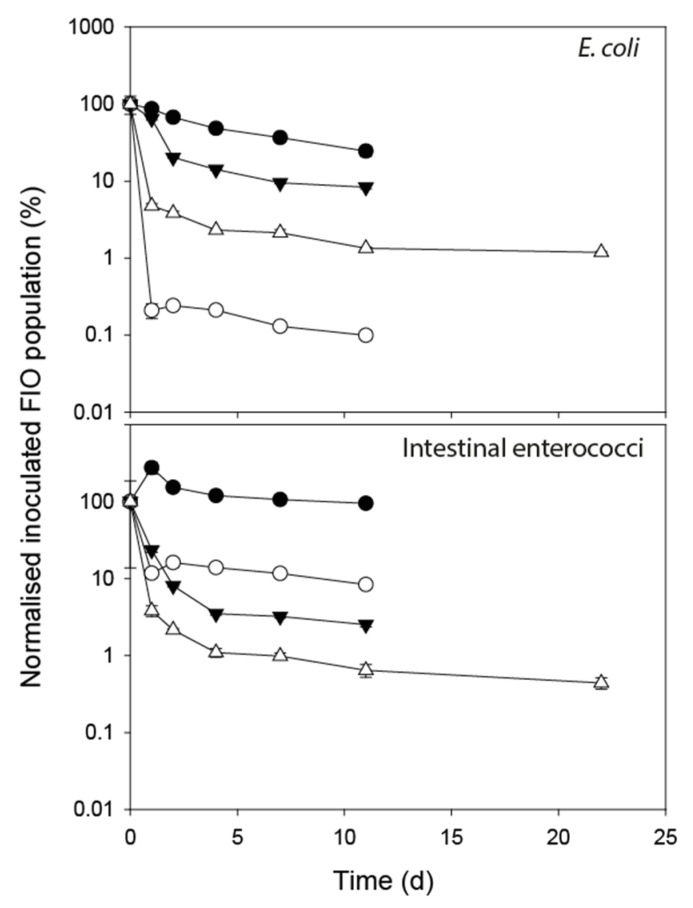
Normalised die-off profiles of faecal indicator organisms (FIOs) inoculated into water undergoing freeze–thaw (F–T) cycles: isolated from dairy cow faeces held at −4 °C F–T (solid black circles) and −8 °C F–T (solid black triangle); isolated from red deer faeces held at −4 °C F–T (open circles) and −8 °C F–T (open triangles). Data points show mean of 3 replicates ± standard error.

The change in FIO concentration (normalised to 100% of the inoculum concentration), for all water treatments is shown in Figure 3. The persistence of FIOs in water across all F–T treatments did not follow a consistent die-off pattern and so both non-linear and log-linear models of population decline were fitted to the data.

#### 3.2.1. *E. coli*

Linear regression models were applied to all deer replicates (r^2^ ranged from 0.519 to 0.957) to determine modelled linear decline rate constants and decimal reduction times (*D*-values, Table 4). Both F–T treatments for deer faeces clearly displayed a two phase die-off, with an immediate rapid decline shifting to a slower decline phase after 24 h; however, the non-linear model was a poor fit and so a two-phase log-linear model was applied. The data for both dairy treatments mapped well to the previously described non-linear model: dairy −4 °C F–T (λ = 0.094 day^−1^, A = 6.399 log_10_ CFU mL^−1^, B = 0.954 log_10_ CFU mL^−1^); dairy -8 °C F–T (λ = 0.390 day^−1^, A = 5.694 log_10_ CFU mL^−1^, B = 1.143 log_10_ CFU mL^−1^).

There was no significant difference in λ for *E. coli* in water versus faecal treatments exposed to the same F–T temperature regime. The modelled drop in population numbers was not significantly different for water and dairy faecal treatments exposed to the −4 °C F–T cycle but for the −8 °C F–T cycle the *E. coli* in the dairy faeces experienced the larger population decline (*p* = 0.01). It was not possible to directly compare the deer treatments due to the different model profiles, but the pattern of decline in water versus faecal treatments was similar.

#### 3.2.2. Intestinal Enterococci

A non-linear model (λ = 0.89 day^−1^, A = 7.390 log_10_ CFU mL^−1^, B = 2.122 log_10_ CFU mL^−1^) mapped best to the intestinal enterococci sourced from deer faeces exposed to the -8 °C F–T cycle and likewise the dairy faeces exposed to the -8 °C F–T cycle (λ = 0.57 day^−1^, A = 4.460 log_10_ CFU mL^−1^, B = 1.593 log_10_ CFU mL^−1^). A linear model was more fitting to the cells sourced from deer faeces exposed to the −4 °C F–T cycle (*D* = 25.5 days) while the cells isolated from the dairy faeces and held in water at the −4 °C F–T cycle showed a small increase in concentration, affecting the model fit. The intestinal enterococci sourced from deer and exposed to the −8 °C F–T showed no significant difference in non-linear die-off characteristics between faecal and water treatments, but those sourced from dairy cows and exposed to the −8 °C F–T were found to exhibit a significantly higher λ in water relative to faeces (*p* < 0.05) but maintained an overall modelled population level that was higher than cells in the faecal treatment (*p* < 0.01).

### 3.3. Temperature Fluctuations Within the Faeces

**Figure 4:**
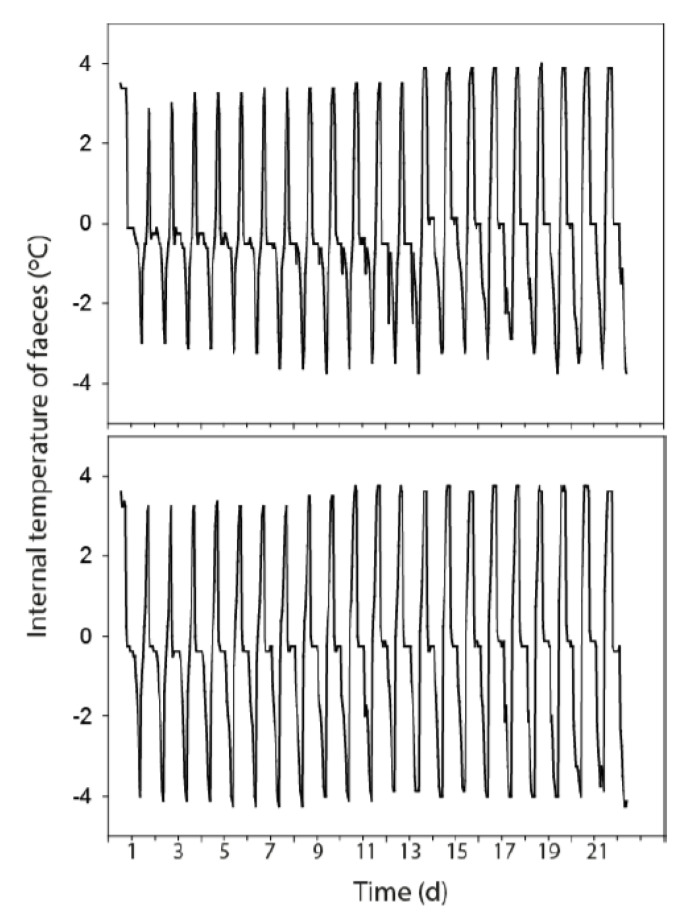
Diurnal patterns of internal temperature of the dairy (**top**) and deer (**bottom**) faecal matrix exposed to the −4 °C freeze–thaw cycle.

The faecal matrix (and therefore associated cells) within the deer faeces appear to be marginally less well insulated from the F–T cycle relative to the dairy faeces, with minimum internal temperatures more frequently approaching that of the external air temperature than observed for dairy faeces (Figure 4). Similar diurnal patterns and trends were observed for the −8 °C F–T cycle.

### 3.4. Changes in Moisture Content

**Figure 5:**
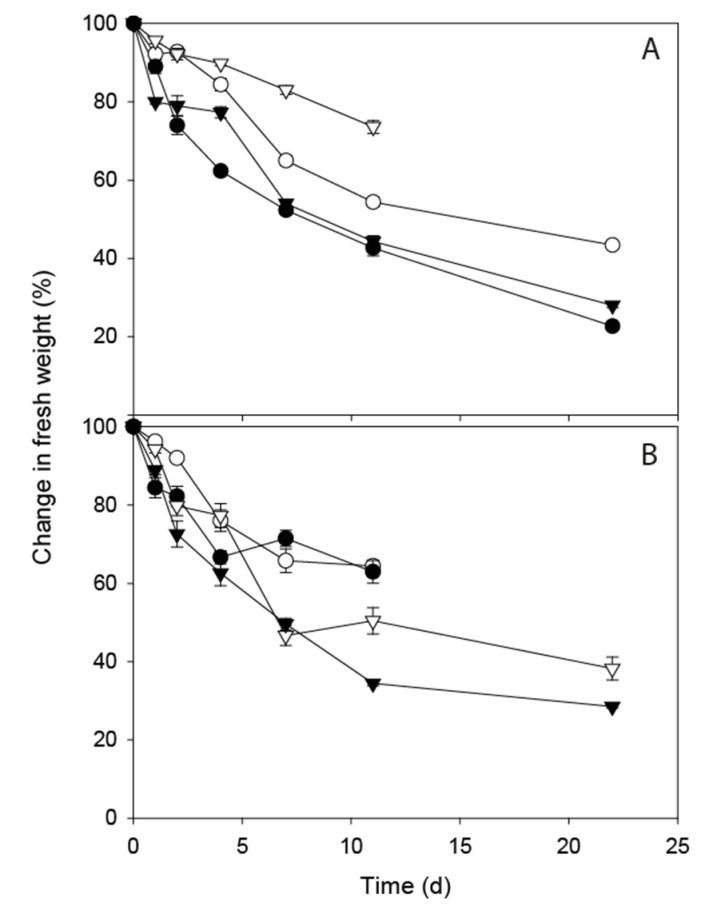
Changes in fresh weight (%) over time dairy faeces (**A**) and deer faeces (**B**) held at: constant 4 °C (solid black circle); −4 °C freeze–thaw (open circles); constant 0 °C (solid triangle); −8 °C freeze–thaw (open triangles). Data points are the mean of five replicates ± standard error.

In dairy faeces, the rate of moisture content loss was more rapid under constant temperature conditions than under F–T cycles (Figure 5). After 22 days, dairy faeces reduced to 22.7% and 28.0% of the original faecal weight (4 °C and 0 °C, respectively), compared to 43.4% and 53.8% (−4 °C F–T and −8 °C F–T cycles, respectively). Fresh weight reduction over time in deer faeces was initially more rapid at 4 °C relative to the −4 °C F–T, but after 11 days both treatments converged to similar fresh weight values (Figure 2). Changes in moisture content followed similar rates of change in both the −8 °C F–T cycle and the constant 0 °C treatments for deer faeces, but after 22 days the 0 °C treatment had dropped to 28.5% of the original weight compared with 38.2% for the −8 °C F–T treatment.

## 4. Discussion

Sub-freezing conditions are often considered hostile to gut-derived bacteria and detrimental to FIO persistence, but quantitative evidence to support our understanding of FIO persistence in the environment in response to different cold temperature regimes remains limited. This study provides novel FIO die-off data in dairy cow and red deer faeces exposed to low temperatures, including freezing conditions and through repeated F–T cycles. A key result is that both *E. coli* and intestinal enterococci are capable of surviving these harsh temperature conditions with the faeces from both animals able to sustain relatively high FIO concentrations, as indicated by modelling, and observations revealing persistence in excess of 11 days and in some cases confirmed beyond 22 days. Faecal contamination of surface waters can pose a public health risk to downstream users, e.g., via recreational exposure [21]. To predict the risk associated with different receiving waters we first need to understand how microbial pollutants, e.g., FIOs, survive in the environment and improve our knowledge of FIO contributions from sources other than just humans and livestock, i.e., quantify FIO risk from wildlife sources. The findings from this series of experiments therefore contribute to an improved understanding of how catchment FIO burden can vary over time, which is fundamentally important for accurate assessment of the risk of microbial contamination of watercourses at the catchment scale [22]. In particular, these data help constrain the parameterisation of die-off coefficients used in the modelling of fate and transfer of FIOs in landscapes where F–T cycling is common (e.g., [23]). The inclusion of laboratory-derived process representation into catchment-scale models needs careful assessment but, given that temperature is such a well-recognised driver of FIO survival, any additional understanding regarding more nuanced temperature-driven FIO responses in both agricultural and wildlife sources is likely to be important for advancing both our predictive capability and appreciation of uncertainty in catchment-scale modelling [7].

The two F–T temperature cycles used in this experiment did not result in different exponential rate constants (λ), which govern the decay of the die-off rate constant over time. FIOs in both treatments would have experienced mechanical disruption to cells, e.g., dehydration and shrinking, as a result of extracellular ice crystal formation typical of the temperature regimes used in this study [24]. The growth characteristics of ice crystals are influenced by the rate of cooling [25], and this rate of temperature change in turn influences cell die-off [26]. In our study, different minimum and maximum temperatures were associated with the two F–T cycles, but the range in temperature was the same for both treatments (8 °C). Cooling rates would therefore have been similar in both treatments, potentially explaining the lack of difference in λ associated with the models of FIO die-off. However, further research on how the magnitude of the F–T fluctuation impacts on FIO persistence in faeces in relation to differential cooling rates is warranted, and sample size in terms of faecal load or water volume is likely to play a role in the rate of cooling and hence population die-off characteristics (e.g., [23]).

Although exponential rate constants did not vary between F–T treatments other survival curve characteristics such as the final level of FIO concentration and the magnitude of drop in FIO population were significantly different. This is important because the % decrease in FIO population per unit time observed in the exponential model jointly reflects λ and B. Dairy faeces sustained FIOs at higher concentrations over the course of repeated F–T cycles relative to deer faeces, highlighting a need for distinct parameter combinations in models of FIO die-off in dairy versus deer faecal sources in the environment. Biphasic decay of FIO populations is increasingly recognised as a consequence of rapid initial die-off of labile cells followed by slower die-off of resistant cells [27]; it may be that the strains of *E. coli* and intestinal enterococci derived from dairy faecal sources were more resistant to temperature stress than strains sourced from deer, with heterogeneity in FIO strain behaviour commonly reported [19]. However, the internal temperature profiles obtained from the faeces provided evidence that the deer faecal pellets were less insulated to the outside air temperature relative to dairy faeces and so a greater proportion of FIOs in deer pellets are likely to have either experienced temperature-induced cell structural damage, or entered a viable-but-non-culturable (VBNC) state, both reducing culturable counts [12,28]. The significant interaction between faecal source and temperature further reinforces the assumption that the structure of a faecal pellet versus a faecal pat is influential on FIO persistence. Moisture loss from faeces via sublimation would become more common with increasing time exposed to sub-zero temperatures and, in general, water content was more efficiently lost from the deer faecal pellets, with this rapid loss of moisture potentially exacerbating FIO die-off further [11].

Of these two FIOs, *E. coli* was more susceptible to die-off when exposed to the F–T cycle with the lower temperature, consistent with enterococci being recognised as a more robust indicator when exposed to freezing stress [23]. Across all treatments, there was very little FIO growth, with temperatures <4 °C known to retard metabolic processes in *E. coli* [19]. A proportion of both FIO cells may have entered into a VBNC state and it may be that some of the differences between the concentrations of *E. coli* and enterococci were attributable to VBNC cells [14,29]. While there is uncertainty over whether cells are therefore truly dead or just metabolically inactive, the ‘die-off’ parameters derived from studies such as ours remain crucially important given that models used to inform on landscape FIO fate and transfer, and guide environmental decision-making, are largely built on data derived from culturable counts in order to align with culture-based standards used by environmental regulators [30].

Our results suggest that die-off responses to just-above-freezing, sub-freezing and F–T temperatures within the range of +4 °C to −8 °C lead to a clear decline in FIO populations. Given that the % decrease in FIO population per unit time jointly reflects λ and B, the differences we determined in parameter B across treatments are crucially important for describing different FIO die-off responses despite values of λ not varying substantially. For example, the magnitude of population decline experienced by *E. coli* in dairy faeces exposed to the −8 °C F–T cycle was more pronounced than when exposed to a constant 0 °C. This difference was only evident for the F–T regime at the lower temperature and likely reflects the greater damage inflicted to cell membranes and walls given that repeated F–T cycles would encourage recrystallisation and therefore promote the growth of larger ice crystals relative to those developing at a constant 0 °C [31]. The difference observed between control and F–T treatments was not apparent in the data for deer faeces, suggesting that the dairy faeces offered greater protection to FIOs at a constant of 0 °C, whereas the detrimental impacts of ice crystal formation appeared to have occurred to a similar extent under both control and F–T treatments for deer faecal pellets. However, at larger scales, many processes will be influenced by a wider set of interacting environmental variables unaccounted for under controlled conditions; those interactions may accelerate or dampen FIO die-off responses to F–T at the range of temperatures investigated and recognising this remains an ever-present challenge of parameterising landscape models with laboratory-derived data [18]. Future investigation of how the specific number of F–T cycles or how varying length of F–T cycles influences survival responses would provide further insight into FIO behaviour under F–T conditions, as would studies of field-relevant die-off under F–T cycles.

Overall, patterns of cell decline were inconsistent in the water mesocosms and required the fitting of different model forms; however, FIO die-off in water relative to faeces exposed to F–T cycles provides useful information for interpreting survival responses, as does the implication of fitting different model forms. The water used in our study was distilled and therefore of a purified form rather than sourced from the environment. When the water mesocosms were exposed to the −4 °C F–T cycle, the water did not freeze, but pure water forms are able to be undercooled by several degrees before ice crystal nucleation initiates [32]. Had an environmental source of water been used, accommodating impurities, particulates and an indigenous microbial community, the nucleation phenomenon may have occurred more quickly due to a more plentiful availability of nucleating centres and potentially impacted on FIO persistence to a greater degree. At −8 °C F–T, the water did freeze but the decline in *E. coli* concentration was more marked in the dairy faeces. This may be due to the fact that the water mesocosms remained frozen and did not thaw under the F–T cycle, whereas the faecal matrix, assumed to offer protection, likely experienced recrystalisation during the F–T cycling, resulting in a more detrimental effect on *E. coli* physiology. This would be consistent with suggestions that cell viability under freezing conditions is influenced more by repeated freeze–thawing than the duration of exposure to freezing conditions [33]. Survival curves of intestinal enterococci sourced from dairy cows and exposed to the lower F–T regime accommodated higher λ values in water relative to faeces but, as with *E. coli*, their final concentration in water was maintained at a higher level than the population held within the faeces. One explanation is that the enterococci, similar to *E. coli*, coped with being encapsulated in ice better than they did in a partially frozen faecal matrix, with population numbers supported by the resistant enterococci subpopulation following the rapid demise of the more labile subpopulation [24,27]. However, the fitting of a log-linear model to relevant data associated with deer-derived FIOs in water highlights an important point. This model indicates that over time FIO concentrations will decay towards zero, whereas the non-linear model used to describe die-off in deer faeces stabilizes above zero, at a value represented by parameter A. If those fitted models held beyond the timespan covered by the experiments, the deer faeces would actually provide protection to the FIOs such that a residual population can be maintained. There is a degree of uncertainty in extrapolating beyond the timespan of the experiment, but the models at least indicate this to be a possibility.

While the deer faeces used in our research was not from truly ‘wild’ deer, or deer farmed over extensive landscape systems, their diet was unlikely to have differed considerably from wild deer. However, one difference might be attributed to a supplementary feed given to the deer through the winter. This feed (palm kernel, wheat feed, beans, barley oat feed cane molasses, calcium carbonate and sodium chloride) amounts to ~0.6 Kg per animal per day; however, wild deer may also supplement their diet by scavenging livestock feeds distributed in farmlands, for example, from feeding troughs [34]. A major gap in our understanding is the behaviour of FIOs sourced from wildlife faeces. Therefore, die-off responses of deer-derived FIOs in both faeces and water exposed to low temperatures provide much needed information to enable better accounting of the varied catchment sources of faecal pollution, and opportunities for model refinement through the inclusion of wildlife sources have been acknowledged (e.g., [35]). Typical values for initial concentrations of FIOs in fresh deer faeces are also valuable for modelling, with source attribution reliant on understanding the relative FIO contributions of wildlife and wildfowl to the landscape [36].

## 5. Conclusions

Understanding *E. coli* and intestinal enterococci die-off in environmental matrices has important implications for managing microbial pollution of wider landscapes. Survival characteristics of FIOs in response to environmental variables such as temperature, UV radiation and moisture availability are used to inform many microbial fate and transfer models and provide evidence to underpin on-farm management practices designed to reduce the risk of microbial transfer from land to aquatic receptors and thus wider contamination of water resources. However, FIO die-off in response to sub-freezing temperatures and F–T cycles in common faecal sources of rural landscapes is not well reported and although investigations of microbial persistence during frozen storage via food microbiology research provide a basis for understanding, it lacks an environmental context. Our results highlight that exposure of fresh faeces to freezing conditions does not completely eradicate the FIO population present and that, in some cases, substantial concentrations of FIOs remain culturable despite lengthy and severe sub-freezing temperatures. Those hardy cells that survive the F–T and sub-freezing temperatures clearly represent a resistant subpopulation, suggesting that if subsequently mobilised from a faecal source and transferred to the wider landscape, their proven ability to persist in the environment may lead to longer-term faecal contamination and greater challenges for environmental regulators. So, while landscape burden of FIOs, whether on-farm or in rural landscapes where deer are common, is likely to be reduced by sub-freezing temperatures it is not an environmental scenario that delivers risk-free and effective removal of FIO source at the landscape-scale. Further, if warmer weather follows soon after F–T cycling, there is potential for a resuscitation of a VBNC population too. Future research should determine how F–T and freezing temperatures affect FIO mobilisation from different faecal sources, including that of wildlife, and whether prior exposure to F–T stress can enhance future resistance and survivability of FIOs as they transfer into different environments.

## Figures and Tables

**Table 1:** FIO concentrations in fresh faeces from the dairy cow and red deer sources used in this experiment.

	FIO Concentration in Fresh Faeces (CFU g^−1^ Dry Weight)			
	*Escherichia coli*		Intestinal Enterococci	
FIO Source	Mean	SE	Mean	SE
Dairy cow	6.49	0.04	6.20	0.05
Red deer	5.30	0.15	5.08	0.06

FIO = faecal indicator organism; CFU = colony forming unit; SE = standard error.

**Table 2:** Parameter values for *E. coli* die-off associated with non-linear models.

Treatment	Exponential Rate Constant λ (Day^−1^)		Level of Population Stability A (log_10_ CFU g^−1^)		Magnitude of Population Decline B (log_10_ CFU g^−1^)	
	Mean	SE	Mean	SE	Mean	SE
Dairy, Freeze-thaw (4, 0, −4 °C)	0.166	0.028	3.757	0.164	2.692	0.172
Dairy, Freeze-thaw (0, −4, −8 °C)	0.410	0.093	2.415	0.266	3.957	0.344
Dairy, constant (4 °C)	0.157	0.023	3.995	0.144	2.572	0.146
Dairy, constant (0 °C)	0.166	0.034	4.504	0.144	1.877	0.149
Deer, Freeze-thaw (4, 0, −4 °C)	0.215	0.089	3.035	0.274	1.696	0.249
Deer, Freeze-thaw (0, −4, −8 °C)	0.122	0.042	1.658	0.701	3.661	0.653
Deer, constant (4 °C)	n/a	n/a	n/a	n/a	n/a	n/a
Deer, constant (0 °C)	0.075	0.019	0.688	0.615	4.630	0.580

n/a = inappropriate model fit.

**Table 3:** Parameter values for intestinal enterococci die-off associated with non-linear models.

Treatment	Exponential Rate Constant λ (Day^−1^)		Level of Population Stability A (log_10_ CFU g^−1^)		Magnitude of Population Decline B (log_10_ CFU g^−1^)	
	Mean	SE	Mean	SE	Mean	SE
Dairy, Freeze-thaw (4, 0, −4 °C)	0.091	0.029	4.738	0.208	1.402	0.189
Dairy, Freeze-thaw (0, −4, −8 °C)	0.314	0.067	2.993	0.165	2.316	0.181
Dairy, constant (4 °C)	n/a	n/a	n/a	n/a	n/a	n/a
Dairy, constant (0 °C)	0.360	0.040	3.981	0.064	2.171	0.086
Deer, Freeze-thaw (4, 0, −4 °C)	0.206	0.093	3.525	0.280	1.512	0.256
Deer, Freeze-thaw (0, −4, −8 °C)	0.121	0.026	1.864	0.282	3.232	0.269
Deer, constant (4 °C)	0.155	0.073	3.980	0.233	1.002	0.216
Deer, constant (0 °C)	0.085	0.027	1.586	0.557	3.527	0.525

n/a = inappropriate model fit.

**Table 4:** Linear decline parameters and decimal reduction times for *E. coli* isolated from deer faeces inoculated into water undergoing freeze-thaw cycling.

Treatment	Modelled Linear Decline Rate (Day^−1^) ^a^	*D*-Values (Days)	R^2^
Deer, Freeze-thaw (4, 0, −4 °C) ^b^	6.209 & 0.086	26.8 ^c^	0.714 ^c^
Deer, Freeze-thaw (0, −4, −8 °C) ^b^	2.957 & 0.06	37.2 ^c^	0.749 ^c^

^a^ = Linear decline rate constant = (2.303 × Figure 3 slope gradient) ^b^ = treatment split into a 2-stage linear decline (rapid and slow) ^c^ = values for “stage 2” slow decline.

## References

[1] Oliver D.M., Bartie P.J., Heathwaite A.L., Reaney S.M., Parnell J.A., Quilliam R.S. (2018). A catchment-scale model to predict spatial and temporal burden of *E. coli* on pasture from grazing livestock. Sci. Total Environ..

[2] Sharma M., Millner P.D., Hashem F., Vinyard B.T., East C.L., Handy E.T., White K., Stonebraker R., Cotton C.P. (2019). *E. coli* survival duration in manure-amended soils is affected by spatiotemporal, agricultural, and weather factors: A multi-season, multi-site field study in the Mid-Atlantic US. Appl. Environ. Microbiol..

[3] García-Aljaro C., Blanch A.R., Campos C., Jofre J., Lucena F. (2019). Pathogens, faecal indicators and human-specific microbial source-tracking markers in sewage. J. Appl. Microbiol..

[4] Kay D., Crowther J., Fewtrell L., Francis C.A., Hopkins M., Kay C., McDonald A.T., Stapleton C.M., Watkins J., Wilkinson J. (2008). Quantification and control of microbial pollution from agriculture: A new policy challenge?. Environ. Sci. Policy.

[5] Cho S., Jackson C.R., Frye J.G. (2020). The prevalence and antimicrobial resistance phenotypes of *Salmonella*, *Escherichia coli*, and Enterococcus sp. in surface water. Lett. Appl. Microbiol..

[6] Jeong J., Wagner K., Flores J.J., Cawthon T., Her Y., Osorio J., Yen H. (2019). Linking watershed modeling and bacterial source tracking to better assess *E. coli* sources. Sci. Total Environ..

[7] Cho K.H., Pachepsky Y.A., Oliver D.M., Muirhead R.W., Park Y., Quilliam R.S., Shelton D.R. (2016). Modeling fate and transport of fecally-derived microorganisms at the watershed scale: State of the science and future opportunities. Water Res..

[8] Oliver D.M., Bird C., Burd E., Wyman M. (2016). Quantitative PCR profiling of *Escherichia coli* in livestock feces reveals increased population resilience relative to culturable counts under temperature extremes. Environ. Sci. Technol..

[9] Smith J.E., Stocker M.D., Hill R.L., Pachepsky Y.A. (2019). The effect of temperature oscillations and sediment texture on fecal indicator bacteria survival in sediments. Water Air Soil Pollut..

[10] Hellberg R.S., Chu E. (2016). Effects of climate change on the persistence and dispersal of foodborne bacterial pathogens in the outdoor environment: A review. Crit. Rev. Microbiol..

[11] Porter K.D., Quilliam R.S., Reaney S.M., Oliver D.M. (2019). High resolution characterisation of *E. coli* proliferation profiles in livestock faeces. Waste Manag..

[12] Wang X., Zhang D., Chen W., Tao J., Xu M., Guo P. (2019). Effects of fulvic acid and fulvic ions on *Escherichia coli* survival in river under repeated freeze-thaw cycles. Environ. Pollut..

[13] Asadishad B., Ghoshal S., Tufenkji N. (2013). Role of cold climate and freeze–thaw on the survival, transport, and virulence of *Yersinia enterocolitica*. Environ. Sci. Technol..

[14] Rocard J.M., Asadishad B., Samonte P.R.V., Ghoshal S., Tufenkji N. (2018). Natural freeze-thaw cycles may increase the risk associated with *Salmonella* contamination in surface and groundwater environments. Water Res. X.

[15] Adhikari H., Barnes D.L., Schiewer S., White D.M. (2007). Total coliform survival characteristics in frozen soils. J. Environ. Eng..

[16] Oliver D.M., Page T., Zhang T., Heathwaite A.L., Beven K., Carter H., McShane G., Keenan P.O., Haygarth P.M. (2012). Determining *E. coli* burden on pasture in a headwater catchment: Combined field and modelling approach. Environ. Int..

[17] Jeon D.J., Ligaray M., Kim M., Kim G., Lee G., Pachepsky Y.A., Cha D.H., Cho K.H. (2019). Evaluating the influence of climate change on the fate and transport of fecal coliform bacteria using the modified SWAT model. Sci. Total Environ..

[18] Oliver D.M., Porter K.D., Pachepsky Y.A., Muirhead R.W., Reaney S.M., Coffey R., Kay D., Milledge D.G., Hong E., Anthony S.G. (2016). Predicting microbial water quality with models: Over-arching questions for managing risk in agricultural catchments. Sci. Total Environ..

[19] Guber A.K., Fry J., Ives R.L., Rose J.B. (2015). *Escherichia coli* survival in, and release from, white-tailed deer feces. Appl. Environ. Microbiol..

[20] Wilkinson J.M., Lee M.R., Rivero M.J., Chamberlain A.T. (2020). Some challenges and opportunities for grazing dairy cows on temperate pastures. Grass Forage Sci..

[21] Russo G.S., Eftim S.E., Goldstone A.E., Dufour A.P., Nappier S.P., Wade T.J. (2020). Evaluating health risks associated with exposure to ambient surface waters during recreational activities: A systematic review and meta-analysis. Water Res..

[22] Neill A.J., Tetzlaff D., Strachan N.J., Hough R.L., Avery L.M., Kuppel S., Maneta M.P., Soulsby C. (2020). An agent-based model that simulates the spatio-temporal dynamics of sources and transfer mechanisms contributing faecal indicator organisms to streams. Part 1: Background and model description. J. Environ. Manag..

[23] Neill A.J., Tetzlaff D., Strachan N.J., Soulsby C. (2019). To what extent does hydrological connectivity control dynamics of faecal indicator organisms in streams? Initial hypothesis testing using a tracer-aided model. J. Hydrol..

[24] Gao W., Leung K., Hawdon N. (2009). Freezing inactivation of *Escherichia coli* and Enterococcus faecalis in water: Response of different strains. Water Environ. Res..

[25] Marcellini M., Noirjean C., Dedovets D., Maria J., Deville S. (2016). Time-Lapse, in situ imaging of ice crystal growth using confocal microscopy. ACS Omega.

[26] Gao W., Smith D.W., Li Y. (2006). Natural freezing as a wastewater treatment method: *E. coli* inactivation capacity. Water Res..

[27] Brouwer A.F., Eisenberg M.C., Remais J.V., Collender P.A., Meza R., Eisenberg J.N. (2017). Modeling biphasic environmental decay of pathogens and implications for risk analysis. Environ. Sci. Technol..

[28] Orruño M., Kaberdin V.R., Arana I. (2017). Survival strategies of *Escherichia coli* and *Vibrio* spp.: Contribution of the viable but nonculturable phenotype to their stress-resistance and persistence in adverse environments. World J. Microbiol. Biotechnol..

[29] Wei C., Zhao X. (2018). Induction of viable but nonculturable *Escherichia coli* O157: H7 by low temperature and its resuscitation. Front. Microbiol..

[30] Oliver D.M., Heathwaite A., Haygarth P.M. (2010). A ‘culture’ change in catchment microbiology?. Hydrol. Process..

[31] Moon H.J., Lee J.Y., Lim J.Y., Kim S.J., Song K.Y., Yoon K.S. (2020). The fate of cold-stressed or tetracycline-resistant *Vibrio* spp. in precooked shrimp during frozen storage. J. Food Saf..

[32] Akyurt M., Zaki G., Habeebullah B. (2002). Freezing phenomena in ice–water systems. Energy Convers. Manag..

[33] Sleight S.C., Wigginton N.S., Lenski R.E. (2006). Increased susceptibility to repeated freeze-thaw cycles in *Escherichia coli* following long-term evolution in a benign environment. BMC Evol. Biol..

[34] Walter W.D., Anderson C.W., Smith R., Vanderklok M., Averill J.J., Ver Cauteren K.C. (2012). On-farm mitigation of transmission of tuberculosis from white-tailed deer to cattle: Literature review and recommendations. Vet. Med. Int..

[35] Neill A.J., Tetzlaff D., Strachan N.J., Hough R.L., Avery L.M., Maneta M.P., Soulsby C. (2020). An agent-based model that simulates the spatio-temporal dynamics of sources and transfer mechanisms contributing faecal indicator organisms to streams. Part 2: Application to a small agricultural catchment. J. Environ. Manag..

[36] Muirhead R.W., Elliott A.H., Monaghan R.M. (2011). A model framework to assess the effect of dairy farms and wild fowl on microbial water quality during base-flow conditions. Water Res..

